# Pathophysiological changes of the lower urinary tract behind voiding dysfunction in streptozotocin-induced long-term diabetic rats

**DOI:** 10.1038/s41598-020-61106-y

**Published:** 2020-03-06

**Authors:** Kazuki Masuda, Naoki Aizawa, Daiji Watanabe, Takatsugu Okegawa, Haruki Kume, Yasuhiko Igawa, Hiroshi Fukuhara

**Affiliations:** 1 Department of Urology, Kyorin University School of Medicine, Tokyo, Japan; 2 Department of Continence Medicine, The University of Tokyo Graduate School of Medicine, Tokyo, Japan; 3 Department of Pharmacology and Toxicology, Dokkyo Medical University School of Medicine, Tochigi, Japan; 4 Department of Urology, The University of Tokyo Graduate School of Medicine, Tokyo, Japan; 5 Department of Urology, Nagano Prefectural Shinshu Medical Center, Suzaka, Japan

**Keywords:** Physiology, Urology

## Abstract

We evaluated pathophysiological characteristics of the lower urinary tract dysfunction in a streptozotocin (STZ)-induced diabetic rat model. STZ (60 mg/kg) was injected intraperitoneally into male Wistar rats. *In vitro* bladder muscle strip experiments, *in vivo* cystometry, and simultaneous recordings of bladder pressure + urethral perfusion pressure (BP + UPP) with or without intravenous administration of L-arginine (300 mg/kg) or tadalafil (0.03 mg/kg) were performed at several time points. *In vitro* muscle strip experiments demonstrated that diabetic rats had significantly higher contractile responses to carbachol at 4–16 weeks, and a tendency for higher contractile responses to electrical field stimulation at 4–12 weeks, but this was reversed at 16 weeks. Diabetic rats had significant increases in voided volume, residual volume, bladder capacity, maximal voiding pressure, and amplitude and frequency of non-voiding contractions at 16 weeks. Tadalafil decreased the residual volume in diabetic rats. Diabetic rats had significantly higher UPP nadir and mean UPP during high-frequency oscillation at 16 weeks, which were reversed by tadalafil or L-arginine administration. The present results suggest that urethral relaxation failure, probably related to impairment of the NO/cGMP signalling pathway, rather than bladder contractile dysfunction may be a prominent cause for voiding dysfunction in STZ-induced chronic diabetic rats.

## Introduction

Underactive bladder (UAB) is defined as a symptom complex suggestive of detrusor underactivity that is usually characterized by prolonged urination time with or without a sensation of incomplete bladder emptying, usually with hesitancy, reduced sensation on filling, and slow stream^1^. Diabetes mellitus (DM) is one of the common causes of UAB. Streptozotocin (STZ) is widely used for the induction of DM in rodents^2^, although it represents type 1 DM. Diabetic bladders may undergo a transition from a compensated to a decompensated state, which begins between 9 and 12 weeks after STZ injection^3^.

As summarized by a systematic review by Ellenbroek *et al*.^4^, however, the majority of previous studies using *in vivo* cystometric measurements found that increases in bladder capacity and PVR in animal models of diabetes are not accompanied by impaired bladder contractility in cystometric or organ bath studies, that is, occur mostly in the absence of detrusor underactivity, while the increase in PVR in diabetic patients is considered to reflect detrusor underactivity. It is not fully clear why increased PVR in patients appears due to detrusor underactivity, whereas such underactivity is typically not observed in animals with increased PVR.

Moreover, diabetes is presumed to impair bladder and urethral function, and urethral relaxation is modulated by the nitric oxide/cyclic guanosine monophosphate (NO/cGMP) signalling pathway^5,6^. A previous study showed urethral relaxant dysfunction in an early phase (5 weeks) of STZ-induced DM rats^7^. In addition, a recent study demonstrated that tadalafil, a phosphodiesterase type 5 (PDE5) inhibitor, improved urethral dysfunction in an early phase (7 weeks) of DM rats^8^. These findings led us a hypothesis that the major cause of voiding dysfunction observed even in a later phase of STZ-induced DM rats should be urethral relaxation failure but not simply detrusor underactivity (decreased bladder contractility). Furthermore, research on diabetic dysfunctions of the bladder and urethra are still limited and controversial; therefore, precise evaluations of the time-dependent changes of bladder-and urethral-functions in DM progression, and the related mechanisms are needed.

In the present study, in order to verify the hypothesis described above, first we assessed time-dependent changes in bladder contractile functions in STZ-induced DM rats. Second, we investigated whether urethral relaxant dysfunction was also present in chronic DM rats and examined the potential involvement of the NO/cGMP signalling pathway in this urethral dysfunction with administration of L-arginine or tadalafil, a PDE5 inhibitor.

## Results

DM rats had significantly higher serum glucose levels and bladder weight, and lower body weight compared with Sham rats at all time points (Table 1).

**Table 1:** Characteristics of rats at 4–16 weeks after streptozotocin or vehicle injection.

	weeks	Sham rats (N)	DM rats (N)
Body weight (g)	4	310 ± 5.6 (5)	189 ± 10**(6)
	8	340 ± 5.3 (7)	166 ± 6.0**(7)
	12	367 ± 8.1 (6)	182 ± 11**(6)
	16	382 ± 4.7 (24)	172 ± 5.9**(24)
Serum glucose (mg/dL)	4	86 ± 1.8 (5)	407 ± 13**(6)
	8	95 ± 3.5 (7)	380 ± 17**(7)
	12	100 ± 5.2 (6)	365 ± 14**(6)
	16	103 ± 3.6 (24)	374 ± 10**(24)
Bladder weight (mg)	4	64 ± 3.8 (5)	115 ± 4.8**(6)
	8	67 ± 2.2 (7)	109 ± 3.6**(7)
	12	62 ± 2.8 (6)	124 ± 5.5**(6)
	16	96 ± 5.3 (24)	159 ± 7.5**(24)

Values are expressed as the mean ± SEM.

N: the number of rats per group.

**P < 0.01: significant difference compared with Sham rats (Mann-Whitney *U*-test).

### *In vitro* muscle strip experiments at 4, 8, 12, and 16 weeks

**Figure 1:**
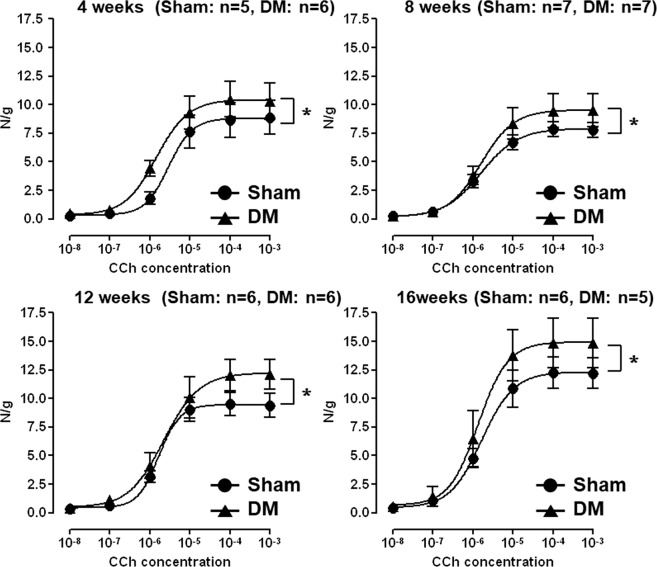
Contractile responses to CCh at 4–16 weeks. Values are expressed as the mean ± SEM. *P < 0.05: Sham vs DM rats (f-test of nonlinear regression).

**Figure 2:**
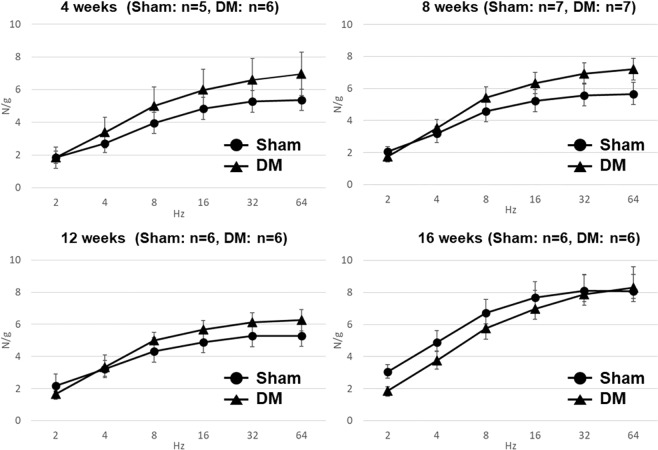
Contractile responses to EFS at 4–16 weeks. Values are expressed as the mean ± SEM. No significant differences were found between Sham and DM rats at all frequencies of EFS at all time-points (Mann-Whitney *U*-test).

Contractile responses to high K^+^ were not significantly different between Sham and DM rats at all time points (Sham: 6.16 ± 0.51 N/g vs DM: 7.08 ± 0.66 N/g at 4 weeks; Sham: 6.03 ± 0.30 N/g vs DM: 6.46 ± 0.36 N/g at 8 weeks; Sham: 7.88 ± 0.56 N/g vs DM: 8.08 ± 0.56 N/g at 12 weeks; Sham: 9.66 ± 0.73 N/g vs DM: 9.16 ± 0.86 N/g at 16 weeks). However, contractile responses to carbachol (CCh) were significantly higher in DM rats compared with Sham rats at all time points (Fig. 1). In addition, DM rats showed a tendency for higher contractile responses to electric field stimulation (EFS) at 4, 8, and 12 weeks, but that was reversed at 16 weeks (Fig. 2). The contraction responses to EFS after α, β-methylene adenosine 5′-triphosphate (mATP), atropine, and tetrodotoxin (TTX) administration were not significantly different between Sham and DM groups at all time points (data not shown).

### Histological examinations at 16 weeks

**Figure 3:**
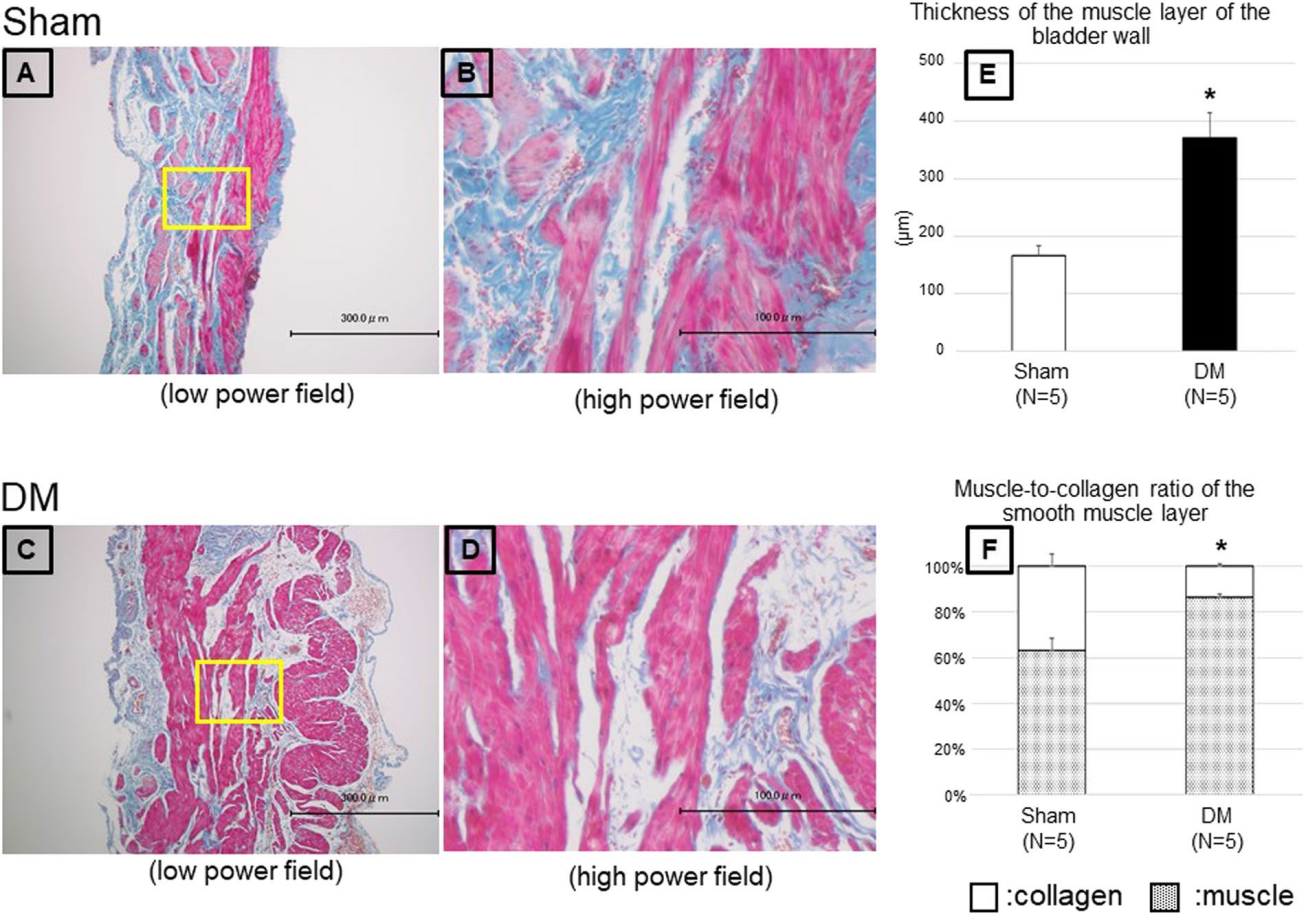
Representative microscopic images with Masson-trichrome staining of the bladder in Sham (**A,B**) and DM (**C,D**) rats (**A** and **C**: low power field, **B** and **D**: high power field). Each square in panels A and C corresponds to panels B and D, respectively. Thickness of the muscle layer in the bladder wall (**E**). Muscle-collagen ratio in the smooth muscle layer (**F**). *P < 0.05: significant differences between Sham rats (Mann-Whitney *U*-test).

The bladder tissues of DM rats showed significant thickening of the detrusor muscle layer compared with those of Sham rats (Fig. 3A–E). In addition, the muscle-to-collagen ratio of the bladder smooth muscle layer was significantly higher in DM rats than Sham rats (Fig. 3F).

### *In vivo* single cystometry (CMG) at 16 weeks

**Figure 4:**
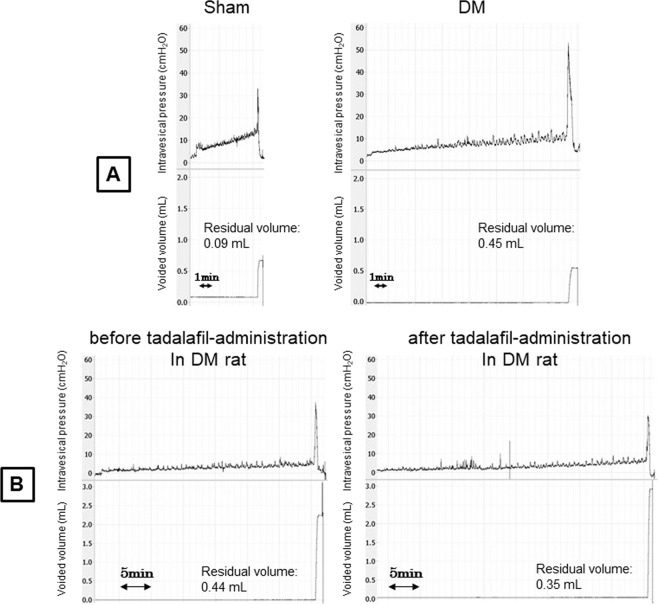
Representative traces of a single CMG recording. (**A**) Representative traces of a single CMG recording in Sham and DM rats at 16 weeks after the induction of DM by STZ. (**B**) Representative traces of a single CMG recording before and after tadalafil administration in a rat 16 weeks after DM induction by STZ.

DM rats showed significant increases in voided volume, residual volume, bladder capacity, opening pressure, maximal voiding pressure, and the amplitude and frequency of non-voiding contractions (NVCs) compared with Sham rats (Fig. 4A, Table 2A). In contrast, voiding efficiency tended to decrease, but it did not reach statistical significance. In separate DM rats, tadalafil (0.03 mg/kg) administered intravenously (i.v.) significantly restored the increased residual volume and the decreased voiding efficiency, but opening pressure and maximal voiding pressure were not significantly different compared with before-administration (Fig. 4B, Table 2B).

**Table 2:** CMG parameters in Sham and diabetic rats at 16 weeks after the induction of DM (A), and before and after tadalafil-administration in DM rats (B).

		Voided volume (ml)	Residual volume (ml)	Bladder capacity (ml)	Voiding efficiency (%)	Threshold pressure for inducing micturition (cmH_2_O)	Opening pressure (cmH_2_O)	Maximal voiding pressure (cmH_2_O)	Basal Pressure (cmH_2_O)	Amplitude of NVCs (cmH_2_O)	Frequency of NVCs (times / min)
A	Sham baseline (N = 8)	0.43 ± 0.04	0.10 ± 0.02	0.54 ± 0.04	80.6 ± 3.45	9.94 ± 1.67	18.9 ± 2.38	34.5 ± 4.44	6.37 ± 1.76	2.96 ± 0.22	0.96 ± 0.15
	DM baseline (N = 9)	1.10 ± 0.18**	0.31 ± 0.11*	1.41 ± 0.18**	76.9 ± 6.20	7.5 ± 0.71	38.2 ± 9.56**	49.9 ± 2.69*	2.95 ± 0.60	4.98 ± 0.31*	1.53 ± 0.23**
B	DM baseline (N = 8) (before i.v.-administration)	1.93 ± 0.24	0.51 ± 0.13	2.44 ± 0.27	78.8 ± 4.52	7.72 ± 1.13	29.3 ± 1.67	36.5 ± 1.19	2.84 ± 0.69	4.70 ± 0.39	1.20 ± 0.19
	DM tadalafil (after i.v.-administration)	1.84 ± 0.25	0.29 ± 0.10^#^	2.13 ± 0.21	85.3 ± 5.56^#^	7.21 ± 1.49	27.0 ± 2.33	42.7 ± 3.50	2.79 ± 0.60	4.56 ± 0.42	1.16 ± 0.15

Values are expressed as the mean ± SEM.

*P < 0.05, **P < 0.01: significant differences compared with Sham baseline (Mann-Whitney *U*-test).

^#^P < 0.05: significant difference compared with DM baseline (paired Student’s *t*-test).

### *In vivo* simultaneous recordings of bladder pressure under isovolumetric conditions and urethral perfusion pressure (UPP) at 16 weeks

**Figure 5:**
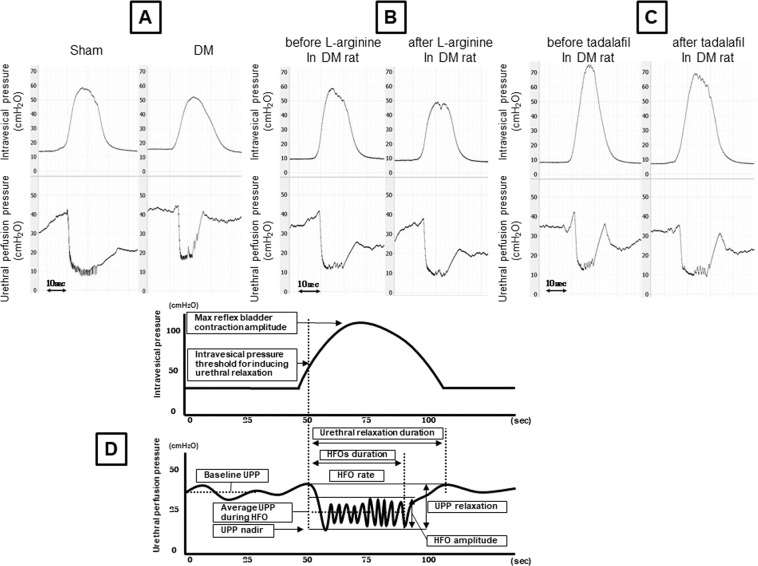
Representative traces of UPP measurements in Sham and DM rats induced by STZ at 16 weeks (**A**), and before and after the administration of L-arginine (**B**), or tadalafil (**C**). (**D**) Schematic diagram showing the parameters of bladder pressure under isovolumetric conditions and urethral perfusion pressure (slightly modified with reference to Torimoto *et al*.^7,23^). HFO: high-frequency oscillation, UPP: urethral perfusion pressure.

DM rats had significantly higher UPP nadir and mean UPP during high-frequency oscillation (HFO) compared with Sham rats (Fig. 5A, Table 3A).

**Table 3:** Parameters of simultaneous recordings of bladder pressure + urethral perfusion pressure (BP + UPP) in Sham and diabetic rats at 16 weeks after induction of DM (A), and before and after L-arginine-(B), or tadalafil-(C) in DM rats.

		Baseline UPP (cmH_2_O)	UPP relaxation (cmH_2_O)	UPP nadir (cmH_2_O)	Urethral relaxation duration (s)	Average UPP during HFO (cmH_2_O)	HFO amplitude (cmH_2_O)	HFO duration (s)	HFO rate (Hz)	Intravesical pressure threshold for inducing urethral relaxation (cmH_2_O)	Max reflex bladder contraction amplitude (cmH_2_O)
A	Sham baseline (N = 6)	29.4 ± 3.75	26.0 ± 3.47	7.93 ± 1.19	28.5 ± 2.11	11.2 ± 1.06	3.24 ± 0.44	15.9 ± 2.39	3.17 ± 0.35	21.2 ± 2.02	60.7 ± 5.13
	DM baseline (N = 6)	40.4 ± 4.20	30.2 ± 4.72	13.1 ± 1.14*	34.2 ± 3.35	20.2 ± 1.21**	2.72 ± 0.28	17.3 ± 2.54	2.86 ± 0.28	19.3 ± 3.00	69.9 ± 9.20
B	DM baseline (N = 6) (before i.v.-administration)	27.6 ± 3.95	20.8 ± 4.04	12.8 ± 1.65	21.9 ± 2.37	18.0 ± 1.67	2.01 ± 0.13	11.0 ± 1.18	3.43 ± 0.47	24.2 ± 5.10	55.2 ± 1.94
	DM L-arginine (after i.v.-administration)	24.4 ± 2.95	18.8 ± 2.41	11.5 ± 1.70^#^	22.6 ± 1.94	16.2 ± 1.59^#^	1.92 ± 0.13	11.5 ± 1.08	3.50 ± 0.50	21.5 ± 3.55	50.9 ± 2.29
C	DM baseline (N = 6) (before i.v.-administration)	31.1 ± 4.76	25.9 ± 3.63	9.42 ± 1.45	31.6 ± 4.74	18.4 ± 3.77	2.60 ± 0.59	14.4 ± 2.12	3.01 ± 0.36	18.0 ± 1.58	58.7 ± 3.74
	DM tadalafil (after i.v.-administration)	30.6 ± 4.69	24.6 ± 4.15	9.11 ± 1.11	31.2 ± 3.74	14.8 ± 2.76^#^	2.66 ± 0.74	13.8 ± 1.61	3.17 ± 0.36	16.5 ± 2.20	56.5 ± 2.70

Values are expressed as the mean ± SEM.

*P < 0.05, **P < 0.01: significant differences compared with Sham baseline (Mann-Whitney *U*-test).

^#^P < 0.05, ^##^P < 0.01: significant differences compared with DM baseline (each before drug-administration, paired Student’s *t*-test).

In separate DM rats, L-arginine (300 mg/kg, i.v.) significantly decreased UPP nadir and the mean UPP during HFO compared with baseline (Fig. 5B, Table 3B). Tadalafil (0.03 mg/kg, i.v.) significantly decreased the mean UPP during HFO compared with baseline (Fig. 5C, Table 3C). Similar effects of these two drugs were observed in Sham rats, but they did not reach statistical significance (data not shown).

## Discussions

In this study, we evaluated the time-dependent changes in bladder contractile function of STZ-induced DM rats using *in vitro* muscle strip experiments. The results demonstrated higher contractile responses to CCh in DM rats at all time points, suggesting that hypersensitivity of the muscarinic receptors in the detrusor smooth muscle occurred during the early-to-late phases of STZ-induced diabetic bladder. This was consistent with a previous finding^4,9^. However, in contractile responses to EFS, DM rats had increased responses at 4, 8, and 12 weeks, but decreased responses at 16 weeks compared with Sham rats. These results suggest that bladder efferent nerve impairment may start in DM rats at 16 weeks after STZ-injection. A previous study reported that disease progression had a biphasic pattern in a rat model of STZ-induced type I DM; early (1–8 weeks) diabetic bladder dysfunction was characterized by bladder overcompensation, whereas its decompensation occurred in the late stage (after 8 weeks)^3^. Our results may support this hypothesis, and further suggest that decompensation may occur at 16 weeks. Taken together, we conducted further *in vivo* functional investigations at 16 weeks after the induction of DM.

By CMG measurements, DM rats had a larger voided volume, residual volume, and bladder capacity, higher maximal voiding pressure and opening pressure, and increased amplitude and frequency of NVCs. Based on these results, we speculated that the voiding impairment present in rats at 16 weeks after the induction of DM was probably caused by bladder outlet obstruction rather than detrusor underactivity and its related bladder overactivity was represented by the increased NVCs. Similar findings in the development of NVCs were reported in DM rats at 4 and 12 weeks after STZ-injection^10^. In STZ-induced DM rats, Pitre *et al*. demonstrated time-dependent bladder wall remodelling characterized by increased smooth muscle and urothelium and decreased collagen prevalence starting 5 weeks after STZ-injection^11^. Liu and Daneshgari reported similar findings on bladder remodelling observed 2–3 weeks after induction and which remained stable from 3–9 weeks in STZ-induced DM rats^12^. In accord with these previous studies, the DM rats in the current study had obvious bladder remodelling at 16 weeks. Similar bladder remodelling was demonstrated in a rat model of bladder outlet obstruction, where NVCs were prominent^13^. Thus, the development of NVCs in DM rats in the current study may be related to bladder remodelling, but this point needs to be investigated in future studies.

If bladder contractility was impaired at 16 weeks after STZ-injection as supposed by the weaken responses to EFS in the *in vitro* bladder muscle stirp investigation, the maximal voiding pressure in the CMG measurement would be expected to be lower in the DM rats. However, it was in fact opposite. We presumed that the higher maximal voiding pressure and opening pressure demonstrated in the DM rats may be due to urethral relaxant dysfunction during voiding, as this type of urethral dysfunction was reported at an early phase (5–7 weeks) of STZ-induced DM rats^7,8^. To determine whether urethral relaxant dysfunction occurs also in the present (16 weeks STZ-induced) DM rats, we performed simultaneous recordings of bladder pressure under isovolumetric conditions and UPP. We found that DM rats had higher UPP nadir and higher mean UPP during HFO. These results suggested that DM rats had impaired urethral relaxation during voiding at least by 16 weeks after the induction of DM. To examine the mechanisms of the urethral dysfunction in DM rats we investigated the potential contribution of the NO/cGMP signalling pathway, as this pathway was reported to be a primary mechanism for urethral relaxation^7,14–16^. In the single CMG investigation with administration of tadalafil, we found decreased residual volume and improved voiding efficiency after tadalafil-administration, although the maximal voiding pressure and opening pressure did not change significantly, which might be influenced by the lower baseline values compared with those in the first group of DM rats for comparison with sham rats. Nevertheless, the urethral relaxation impairment demonstrated in the simultaneous UPP measurements in the chronic DM rats was reversed after the administration of L-arginine or tadalafil. Torimoto *et al*. reported that diabetic urethral dysfunction associated with NO deficiency occurred at an early phase (at 5 weeks)^7^. Gotoh *et al*. reported that tadalafil restored bladder blood flow and lower urinary tract function, including urethral function, at an early phase of DM (at 7 weeks)^8,17^. Our results suggest that long-term diabetes caused NO/cGMP deficiency, resulting in urethral relaxant dysfunction. Tadalafil-administration in the CMG experiments improved the larger residual volume and the lower voiding efficiency in DM rats, which may be indirectly affected by the activation of NO/cGMP on urethral dysfunction.

Sensory information from the lower urinary tract is conveyed to the spinal cord to trigger and co‐ordinate micturition. Nakagawa *et al*. recently reported that urethral-responsive sensory neurons in STZ-induced DM rats were numerically fewer and exhibited functional loss^18^. In addition, Marini *et al*. reported that STZ-induced DM rats have fibrotic changes on urethral muscle^19^. These neuronal and structural changes of the urethra might contribute to the urethral relaxant dysfunction demonstrated in the present study. It’s most conceivable that urethral relaxation failure during voiding may be the prominent mechanism involved in voiding dysfunction observed in a chronic diabetic rat model induced by STZ.

There were some study limitations. First, we did not characterize DM rats induced by STZ after 16 weeks. Second, we did not perform direct analyses of the NO/cGMP signalling pathway in the present study, such as the quantitative analyses of mRNA and protein expressions of NO synthase isoforms assessed by quantitative PCR, immunohistofluorescence, and Western blots. In addition, we did not investigate a possible contribution of diuresis itself to the pathophysiology of STZ-induced diabetic rats. A further study is needed to investigate these points. To verify the role of the NO/cGMP signalling pathway in causing urethral relaxation failure demonstrated in STZ-induced DM rats, additional experiments with a long-term treatment with tadalafil starting at STZ-injection for preventing the development of the urethral relaxation failure would be valuable.

In conclusion, the current results suggest that urethral relaxation failure, probably related to impairment of the NO/cGMP signalling pathway, rather than bladder contractile dysfunction during voiding is a prominent mechanism involved in voiding dysfunction observed in a chronic diabetic rat model induced by STZ.

## Methods

### Animals and experimental group

A total of 110 adult (9 weeks old) male Wistar rats (Japan SLC, Hamamatsu, Japan) were used. The rats were maintained under standard laboratory conditions with a 12/12 h light/dark cycle and free access to food and water. Experimental protocols were approved by the Institutional Animal Care and Use Committee of the University of Tokyo and were in line with NIH guidelines for the care and use of experimental animals. In the STZ-induced diabetes group, 60 mg/kg STZ dissolved in 0.05 M citrate buffer (pH 4.5) was intraperitoneally injected. Blood samples were collected from the tail vein after 48 h, and rats with a serum glucose level of 300 mg/dl or higher were used. Control rats received vehicle (0.05 M citrate buffer)-injection instead of STZ. In the present study, approximately 30% DM rats were excluded due to either unsuccessful induction of diabetes or death after induction of diabetes. Moreover, DM rats did not receive any insulin injections.

### *In vitro* muscle strip experiments at 4, 8, 12, and 16 weeks

*In vitro* muscle strip experiments using full-thickness longitudinal strips taken from the bladder body were performed at 4, 8, 12, and 16 weeks after the induction of diabetes as described previously^20^. Longitudinal, full-thickness detrusor strips (about 5 × 1 mm) were transferred to 5 ml organ baths filled with oxygenated Krebs solution. The strip was first exposed to a high K^+^-containing (62 mM KCl) Krebs solution. Then, the following stimuli were examined: CCh cumulative administration (10^−8^–10^−3^ M), or EFS (2–64 Hz, 50 V, 0.8 m sec pulse duration, 5 s train duration, 2 min interval). After baseline EFS measurement, contractions were evoked by EFS with cumulative administrations of 10^−5^ M mATP (five times at 2 min intervals), 10^−6^ M atropine administration, and finally 10^−6^ M TTX.

### Histological examinations at 16 weeks

Histological examinations were performed as previously described^13^. In separate animals, the whole bladder was isolated, subsequently fixed in 4% paraformaldehyde-PBS, embedded in paraffin and then cut into 3-μm sections. Masson-trichrome staining was used to evaluate the thickness of the detrusor muscle layer and fibrosis in the detrusor muscle layer and whole bladder wall. The thickness of the detrusor muscle layer was measured in ten randomly selected sections. Collagen-deposition was determined in four high power fields from randomly selected sections. The images were analysed using Adobe and ImageJ software (http://rsb.info.nih.gov/ij/).

### *In vivo* single CMG at 16 weeks

The methods for CMG measurements were slightly modified from our previous study^21^. In brief, under isoflurane anaesthesia, a PE-50 catheter (Clay Adams, Parsippany, NJ, USA) with a cuff was implanted into the bladder through the bladder dome and secured by a purse-string suture with a 5-0 prolene thread. In addition, another PE-50 catheter was placed in the left jugular vein for the tadalafil drug administration trial. Each rat was restrained in a Bollman cage (Type III KN-326; Natsume Co., Ltd, Japan) and allowed to adapt for 2 h prior to performing CMG. Saline was continuously instilled into the bladder at a rate of 6 ml/h. In separate animals, after the baseline measurement, tadalafil (0.03 mg/kg) was intravenously administered, and CMG measurements were repeated at 10 min after drug-administration. The following CMG parameters were analysed: voided volume, residual volume, bladder capacity, voiding efficiency, threshold pressure for inducing micturition, opening pressure, maximal voiding pressure, basal pressure and NVCs. NVCs, defined as bladder contractions with an amplitude greater than 2 cm H_2_O observed during the filling phase, were analysed to calculate their amplitude (cm H_2_O) and frequency (times/min).

### *In vivo* simultaneous recordings of bladder pressure under isovolumetric conditions and UPP at 16 weeks

These measurements were performed as previously described^7,22,23^. Under urethane anaesthesia (1.0 g/kg, subcutaneously), a PE-50 catheter with a cuff was implanted into the bladder side wall and secured by a purse-string suture with a 5-0 prolene thread to monitor intravesical pressure. To monitor UPP, another fabricated double-lumen catheter (PE-160 and PE-50) was implanted into the bladder top by a purse-string suture with a 5-0 prolene thread while pressing the tip into close contact with the bladder neck. To prevent backflow filling with urine from the bladder to the ureters during the experiment, both ureters were ligated. Another catheter (PE-50) was placed in the blood vessel for drug administration.

The urethra was perfused with saline via the outer cannula of the double lumen catheter at 0.075 ml/min using an infusion pump under open urethra conditions. The bladder was filled with saline (6.0 ml/h) until an isovolumetric rhythmic bladder contraction occurred. In separate animals, after baseline measurement for 30 min, tadalafil (0.03 mg/kg) or L-arginine (300 mg/kg) was intravenously administered, and measurements were repeated at 10 min after drug-administration.

The following bladder pressure and UPP parameters were analysed: baseline UPP, UPP relaxation, UPP nadir, urethral relaxation duration, mean UPP during HFO, HFO amplitude, HFO duration, HFO rate, intravesical pressure threshold for inducing urethral relaxation, and max reflex bladder contraction amplitude (Fig. 5D).

### Drugs

STZ, CCh, atropine and TTX were purchased from Wako Pure Chemical Industries, Ltd, Tokyo, Japan. mATP was purchased from Tocris Bioscience, Bristol, UK. Tadalafil was purchased from TRC Canada. L-Arginine was purchased from Sigma-Aldrich St. Louis, MO, USA. Tadalafil was dissolved in *N, N*-dimethylacetamide, and then saline was added to adjust to an appropriate concentration. L-arginine was dissolved in saline.

### Statistical analysis

All data are expressed as the mean ± standard error of mean (SEM). Dose responses for CCh were analysed by f-test of nonlinear regression. Other results were analysed using the Mann-Whitney *U*-test or paired Student’s *t*-test. P < 0.05 was considered statistically significant.
